# A Case of Pediatric Retroperitoneal Schwannoma Presenting with Myoclonus of the Lower Limb: A Case Report and Review

**DOI:** 10.70352/scrj.cr.24-0077

**Published:** 2025-03-11

**Authors:** Takazumi Kato, Yuki Sengoku, Shinya Banno, Souji Ibuka, Saori Endo, Michio Ozeki, Yukiko Tani, Naruhiko Murase, Yuta Sato, Itaru Yasufuku, Yu Jesse Tajima, Nobuhisa Matsuhashi

**Affiliations:** 1 Department of Digestive Surgery and Pediatric Surgery, Gifu University Graduate School of Medicine, Gifu, Gifu, Japan; 2 Department of Pediatrics, Gifu University Graduate School of Medicine, Gifu, Gifu, Japan; 3 Department of Pediatric Surgery, Japanese Red Cross Aichi Medical Center Nagoya Daiichi Hospital, Nagoya, Aichi, Japan

**Keywords:** schwannoma, retroperitoneal tumor, child, myoclonus

## Abstract

**INTRODUCTION:**

Schwannomas arise from Schwann cells of the peripheral nerve sheath. Schwannomas are usually benign, and most of them are located in the head, neck, or distal extremities. The retroperitoneal region is an unusual location for schwannomas. Schwannomas are usually seen in adults and are very rare in the pediatric population.

**CASE PRESENTATION:**

A 6-year-old boy was referred to our institution with a right abdominal mass. His main complaint was intermittent myoclonus of his right lower limb. Abdominal computed tomography (CT) and magnetic resonance imaging scans revealed a round-shaped solid mass measuring 78 × 61 mm adjacent to the caudal side of the right kidney. Right hydronephrosis and hydroureters resulting from ureteral compression were present. A positron emission tomography-CT scan showed mild accumulation of fluorodeoxyglucose. Tumor resection was performed by laparotomy, and the mass was completely excised. Postoperative pathologic examination showed a benign schwannoma. The myoclonus of the right lower limb that had been present before surgery disappeared after surgery. At 9 months since the operation, there has been no recurrence.

**CONCLUSIONS:**

We present a pediatric case of a retroperitoneal schwannoma causing myoclonus of the lower limb. Retroperitoneal schwannomas in children are extremely rare, with only 4 cases having been reported in English.

## Abbreviations

CT
: computed tomography
FDG
: fluorodeoxyglucose
MRI
: magnetic resonance imaging
PET
: positron emission tomography

## INTRODUCTION

Schwannomas arise from Schwann cells of the peripheral nerve sheath, and most schwannomas are located in the head, neck, or distal extremities. The retroperitoneal region is an unusual location for schwannomas, comprising approximately 0.5%–5% of all cases of schwannoma that are retroperitoneal.^1)^ Schwannomas are usually seen in adults between the 2nd and 5th decades of life and are very rare in the pediatric population.^2)^

Complete resection of schwannomas is recommended because these tumors are insensitive to radiotherapy and chemotherapy. Retroperitoneal schwannomas are, on the whole, mostly benign, but malignant tumors have also been reported.^2)^ Here, we present a pediatric case of a retroperitoneal schwannoma causing myoclonus of the lower limb.

## CASE PRESENTATION

A 6-year-old boy was referred to our institution with a right abdominal mass that was detected on abdominal computed tomography (CT) during an investigation into complaints of intermittent myoclonus of his right lower limb. He suddenly developed involuntary movements of the right lower limb (myoclonus with strong flexion of his right thigh) while at elementary school, and he could not stop this symptom. An abdominal examination revealed a softball-sized hard mass in the right abdomen, and an abdominal CT scan was performed. A spherical tumor measuring 75 mm in diameter was found, and he was referred to our hospital for further examination and treatment. There was nothing of note in either his medical or familial history.

**Fig. 1:**
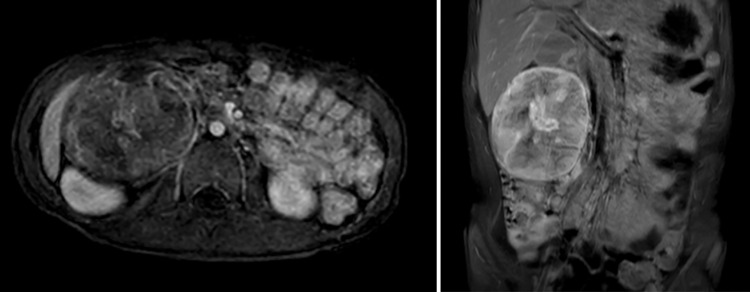
Abdominal MRI with contrast. A solid mass 78 × 61 mm in diameter was identified in the right lower renal region on MRI. The contrast-enhanced MRI scan shows heterogeneous enhancement of the lesion, especially in the central part of the tumor. MRI, magnetic resonance imaging

**Fig. 2:**
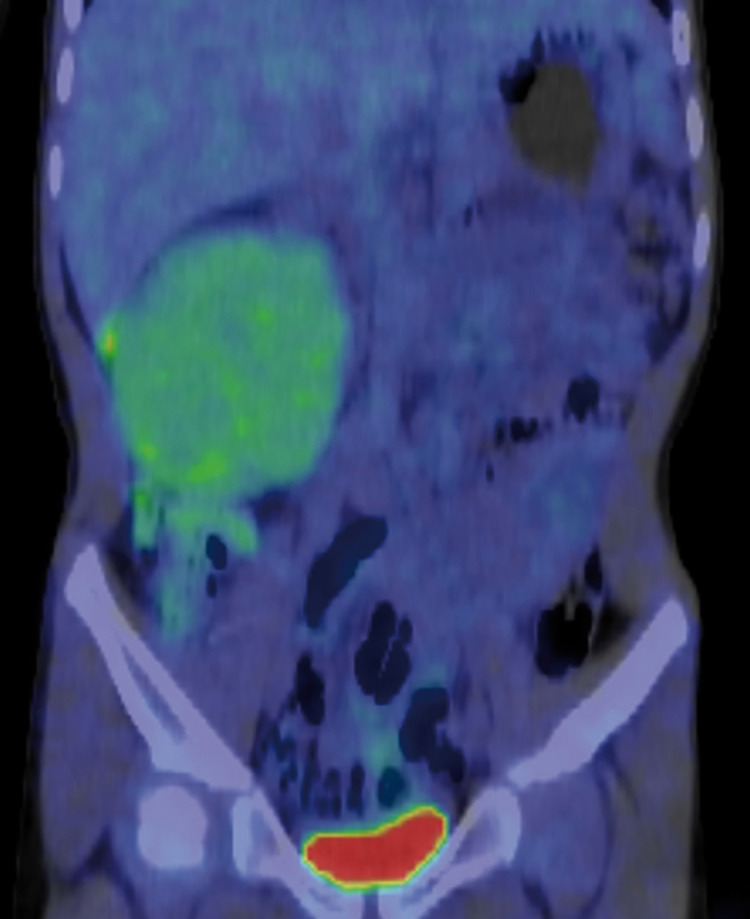
^18^F-FDG-PET CT. The tumor showed slight accumulation of FDG (SUV max2.0), and was considered to be benign or a low-grade malignancy. ^18^F-FDG, ^18^F-fluorodeoxyglucose; CT, computed tomography; PET, positron emission tomography

His height was 115 cm, weight 18.3 kg, and body mass index 13.84 kg/m^2^. Physical examination revealed a mass about the size of an adult fist in his right abdomen. No café-au-lait spots or masses of the skin suggestive of neurofibromatosis 1 were found. The patient’s routine blood tests were normal. Examination of tumor markers showed a neuron-specific enolase level of 40.1 ng/mL and an interleukin-2 receptor (IL2-R) level of 511 U/mL, both of which were slightly high. Homovanillic acid and vanillylmandelic acid in urine were 10.0 µg/mg creatinine (CRE) and 7.8 µg/mg CRE, respectively, and all other markers were normal. Abdominal CT and magnetic resonance imaging (MRI) scans revealed a round-shaped solid mass measuring 78 × 61 mm adjacent to the caudal side of the right kidney (**Fig. 1**). The mass was well defined and showed no signs of infiltration into the surrounding tissues. Right hydronephrosis and hydroureters resulting from ureteral compression were present. The tumor showed contrast enhancement and was nourished by the lumbar artery. There was no swelling of the intraperitoneal lymph nodes. We determined that MIBG scintigraphy was indicated, but positron emission tomography (PET) imaging was used instead because MIBG could not be performed quickly. A PET-CT scan showed mild accumulation of fluorodeoxyglucose (FDG), so radiologically, the tumor was highly suggestive of being benign or a low-grade malignancy, highly negative for neuroblastic tumors, and showed no metastasis (**Fig. 2**).

The preoperative diagnosis was retroperitoneal ganglioneuroma, and surgical tumorectomy was carried out. A stent tube was placed in the right ureter before surgery. Tumor resection was performed by laparotomy. The operation took 4 hours and 13 minutes, with a blood loss of 270 cc. The mass, which was immobile, stiff, and adherent to the surrounding tissue (including the inferior vena cava, right iliopsoas muscle, and right side of the lumbar vertebrae), was completely excised along with part of the fascia of the iliopsoas muscle. The mass was solid, surrounded by a smooth surface capsule, and fortunately had no significant nerve involvement.

**Fig. 3:**
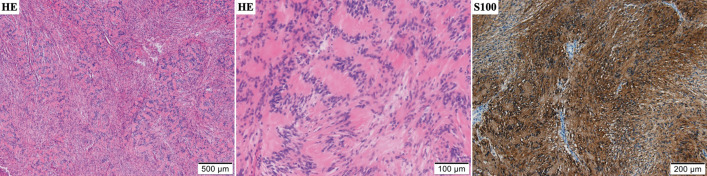
The tumor’s microscopic sections. The solid part of the schwannoma shows proliferation of spindle cells in an eosinophilic spindle cell bundle-like arrangement, including nuclear palisading. Immunohistochemical examination revealed that S100 was strongly positive.

Histopathological findings showed proliferation of eosinophilic spindle cells in a bundle-like arrangement. Nuclear palisading was seen in cellular areas. Immunohistochemical examination revealed that spindle cells were strongly positive for S100 and negative for SMA, desmin, CD34, and EMA (**Fig. 3**). The tumor showed very low mitotic activity and MIB-1 proliferative index was 2%, suggesting a benign tumor. In addition, the expression of H3K27me3, which is often lost in malignant peripheral nerve sheath tumors, was retained. Ganglioneuroma was ruled out because there were no ganglion-like cells. Finally, the patient was diagnosed with a benign schwannoma.

The patient’s clinical course was uneventful, and he was subsequently discharged on postoperative day 6. The myoclonus of the right lower limb that had been present before surgery disappeared after surgery. At 9 months since the operation, there has been no recurrence.

## DISCUSSION

Schwannomas originate from Schwann cells of the peripheral nerve sheath. The affected part of the body generally comprises subcutaneous tissue of the head, neck, or the distal extremities, whereas the retroperitoneal region is not a common location. Retroperitoneal schwannomas account for approximately 0.5%–5.0% of all schwannomas.^1)^ Only patients with von Recklinghausen’s disease have a stronger correlation with retroperitoneal schwannoma,^2)^ but our patient did not have von Recklinghausen’s disease. Schwannomas most frequently occur in adults aged 20–50 years and are extremely rare in children.^2)^ Indicative of the rarity of schwannoma in children, only 12 cases of retroperitoneal schwannomas, including malignant schwannomas, have been reported in the English-language literature, and including our case, only 4 cases of benign schwannomas have been reported (**Table 1**).^1–11)^

**Table 1:** Retroperitoneal schwannoma in children: review of English literature

Author	Publication year	Patient age	Sex	Primary illness	Nature	Follow-up period	Outcome
Kato T	2024	6 years	M	(−)	Benign	9 months	Alive
Wang J^1)^	2018	2 years	M	No description	Malignant	5 months	Alive
Cayirli H^2)^	2016	14 years	M	(−)	Benign	1 year	Alive
Agaimy A^3)^	2014	7 years	F	No description	Malignant	18 years	Alive
Arrabal-Polo MA^4)^	2013	4 years	F	No description	Benign	4 years	Alive
Li Q^5)^	2007	6 months	No description				
Oğuzkurt P^6)^	2001	1 months	M	No description	Malignant	1 year	Alive
Fabbro MA^7)^	1997	11 years	M	No description	Malignant	5 years	Death
Roth MJ^8)^	1993	1 years	F	Congenital pigmented giant nevi	Malignant	8 months	Death
Garcia de Davila MT^9)^	1986	1 years	F	(−)	Malignant	30 months	Alive
Moazam F^10)^	1983	14 years	M	von Recklinghausen’s disease	Malignant	13 weeks	Death
Wilson CS^11)^	1975	2 years	F	No description	Benign	6 years	Alive

Pediatric cases of benign and malignant schwannomas in the retroperitoneum were 12 cases, including our patient. There were only 4 benign schwannomas and 7 malignant schwannomas. Additionally, there is only 1 case with von Recklinghausen’s disease.

The symptoms of a benign schwannoma are usually nonspecific and change according to the location and size of the lesion. Retroperitoneal schwannomas are generally asymptomatic. The most common symptoms are abdominal pain and distention, but other symptoms can include dyspepsia, nausea, colitis, lumbar pain, and urinary symptoms depending on the lesion’s location.^4)^ In our case, the patient presented with myoclonus of the right lower limb due to nerve compression.

Because retroperitoneal schwannomas can mimic many common primary lesions, the preoperative diagnosis of these tumors is challenging and mainly depends on radiological examinations such as CT and MRI. Diagnostically, however, both CT and MRI are nonspecific for retroperitoneal schwannomas. Furthermore, fine-needle aspiration and CT- or ultrasound-guided biopsy have proven ineffective for this tumor because of high cellular pleomorphism and an increased risk of hemorrhage, infection, and tumor seeding.^1)^ In our case, an FDG-PET CT scan was useful in distinguishing between a benign and malignant tumor.

More than 90% of schwannomas are benign, but 1.1%–14.2% may be malignant. Risk factors for malignant transformation include long course, large tumor diameter, rapid growth, and coexistence of neurofibromatosis. The definitive diagnosis is dependent on pathological examination. Although Antoni A and Antoni B areas are seen in various ratios in classic schwannomas, immunohistochemistry of schwannomas shows strong and diffuse staining for S-100, and they are typically negative for CD117, desmin, CD34, HMB-45, synaptophysin, chromogranin, cytokeratin, and smooth muscle actin.^2)^

Because malignancy cannot be excluded with analyses performed pre- or perioperatively, and because schwannomas are insensitive to radiotherapy and chemotherapy, complete resection of the tumor is recommended. However, as schwannomas are benign tumors, although there are concerns about recurrence and growth, partial resection may be an option if total resection would leave neurological sequelae or cause a decrease in quality of life due to damage to surrounding organs. Recent advances in laparoscopic resection and robot-assisted excision have also been reported.^4)^ In addition, if there are no symptoms, untreated follow-up observation is an option. Kitagawa identified 2 types of retroperitoneal schwannoma: those that gradually increased in size and those that did not. They reported a median relative growth rate of retroperitoneal schwannomas of 5.6% per year (range, 0%–31.5% per year), so careful follow-up is necessary.^12)^

In contrast, Benato et al. reported that while en bloc resection may be feasible for visceral tumors, it risks causing permanent neurological deficits in retroperitoneal schwannomas, which are associated with major lumbosacral nerve trunks in more than 20% of cases. The surgical management of retroperitoneal schwannomas is multifaceted and reflects both the complexity of their anatomical location and the risk of neurological damage associated with their resection. The combination of expertise from both neurosurgeons and abdominal surgeons has led to good neurological and oncological outcomes.^13)^ If involvement of the spinal nerve trunks is suspected, preoperative consultation with neurosurgeons would be necessary.

Benign schwannomas have a good prognosis. Recurrence is the most frequent complication, reported in 5%–10% of cases, and is mostly related to incomplete excision.^2)^ Complete resection was performed in our patient, and he has experienced no complications during a 9-month follow-up. Nevertheless, his prognosis is not optimistic, and recurrence is the most serious problem after surgery, making close follow-up absolutely essential.

## CONCLUSIONS

We present a pediatric case of a retroperitoneal schwannoma causing myoclonus of the lower limb. Retroperitoneal schwannomas in children are extremely rare, with only 4 cases having been reported in English. Preoperative diagnosis of retroperitoneal schwannomas is very difficult, but in our case, an FDG-PET CT scan was useful in distinguishing between benign and malignant tumors.

## ACKNOWLEDGMENTS

We would like to thank Tomohiro Kanayama (Department of Tumor Pathology, Gifu University Graduate School of Medicine) who contributed to the histological examination. We would also like to thank Rise Japan LCC (https://rise-japan.rulez.jp/) for English language editing.

## DECLARATIONS

### Funding

None.

### Authors’ contributions

TK drafted the manuscript.

YS, SB, SI, SE, MO, YT, NMu, YS, IY, YJT and NMa provided academic advice.

All authors read and approved the final manuscript.

Accountability for all aspects of the work: all authors.

### Availability of data and materials

The availability of the data used in this case is subject to confirmation by the journal or the authors. For more information on data availability and access procedures, please contact the journal or corresponding author.

### Ethics approval and consent to participate

This case report did not require approval from the institutional ethics committee.

### Consent for publication

Written informed consent was obtained from the patients for publication of this case report and accompanying images.

### Competing interests

The authors declare that they have no competing interests.
